# Basic Exploratory Study of Bisphenol A (BPA) Dietary Administration to Istrian Pramenka Rams and Male Toxicity Investigation

**DOI:** 10.3390/toxics10050224

**Published:** 2022-04-29

**Authors:** Sabina Šturm, Klaus Weber, Primož Klinc, Ellinor Spörndly-Nees, Azadeh Fakhrzadeh, Tanja Knific, Andrej Škibin, Věra Fialová, Yoshimasa Okazaki, Tanja Razinger, Jürgen Laufs, Robert Kreutzer, Milan Pogačnik, Tanja Švara, Vesna Cerkvenik-Flajs

**Affiliations:** 1 Veterinary Faculty, University of Ljubljana, Gerbičeva Ulica 60, 1000 Ljubljana, Slovenia; primoz.klinc@vf.uni-lj.si (P.K.); tanja.knific@vf.uni-lj.si (T.K.); andrej.skibin@vf.uni-lj.si (A.Š.); milan.pogacnik@vf.uni-lj.si (M.P.); tanja.svara@vf.uni-lj.si (T.Š.); vesna.cerkvenikflajs@vf.uni-lj.si (V.C.-F.); 2 AnaPath Services GmbH, Hammerstrasse 49, 4410 Liestal, Switzerland; kweber@anapath.ch (K.W.); yokazaki@anapath.ch (Y.O.); trazinger@anapath.ch (T.R.); jlaufs@anapath.ch (J.L.); rkreutzer@anapath.ch (R.K.); 3 Department of Anatomy, Physiology and Biochemistry, Swedish University of Agricultural Sciences, P.O. Box 7011, 75007 Uppsala, Sweden; ellinor.sporndly-nees@slu.se; 4 Iranian Research Institute for Information Science and Technology (IranDoc) Tehran Province, No. 1090, Enghelab, Tehran 13157 73314, Iran; fakhrzadeh@irandoc.ac.ir; 5 Biopharm, Research Institute of Biopharmacy and Veterinary Drugs, Pohoří-Chotouň 90, 254 01 Jílové u Prahy, Czech Republic; vera.fialova@bri.cz

**Keywords:** bisphenol A, rams, toxicokinetics, male toxicity, sperm

## Abstract

Bisphenol A (BPA), an endocrine-disrupting chemical and environmental pollutant, has been reported by many researchers to induce male reproductive toxicity in different experimental models. In this study, we investigated whether long-term exposure for two months to 25 µg/kg body weight (low dose) of BPA affects spermatogenesis or sperm quality in young Istrian Pramenka rams exposed via diet. We evaluated body and testicular weights, histopathology of testes and epididymides, and sperm analyses, and compared these parameters between the group of treated rams and the control group of rams. Although there were some differences between the two groups, these differences were not large or statistically significant. The only statistically significant difference was the lower epithelial height of seminiferous tubules in treated rams, compared to control rams. In addition to assessing toxicity, BPA concentrations in the blood plasma of treated rams were determined after the first administration, and the toxicokinetic parameters of total BPA were calculated. In this study, no major signs of altered reproduction in rams were detected.

## 1. Introduction

Many synthetic chemicals that are ubiquitous in our environment can have negative effects on the health of wildlife, domestic animals, and humans. Some of them can bind to and disrupt endogenous hormone receptors and are therefore classified as endocrine-disrupting compounds (EDCs) [1].

One of these chemicals is bisphenol A (BPA), a substance produced in large quantities and used in a variety of common consumer products, including food and beverage packaging, flame retardants, adhesives, building materials, electronic components, and paper coatings [2]. The weak estrogenic, anti-androgenic, and anti-thyroid effects of BPA, combined with the increasing incidence of reproductive disorders observed over the past decade, have led to health concerns about human exposure to BPA [3]. Considering health concerns for humans, the European Food Safety Agency (EFSA) established the tolerable daily intake for BPA to 50 μg/kg bw/day, which was subsequently lowered to 4 μg/kg bw/day in 2015 [4]. Furthermore, in the current re-evaluation and draft opinion, the EFSA is considering reducing the TDI even further (0.04 ng/kg bw/day) [5].

To investigate the adverse effects of BPA on the male reproductive tract, numerous in vivo reproductive toxicity studies have been conducted in laboratory rodents [6]. In vivo studies have been conducted primarily during pregnancy or lactation, as these periods appear to be particularly sensitive windows of exposure [7]. Based on occupational exposure of workers from BPA-producing and processing factories, and on epidemiological studies showing the deterioration of semen characteristics in these workers [8], concerns have been raised about whether BPA could affect male reproductive health when exposure occurs during later phases of life, such as puberty or adulthood. Additional in vivo studies have been conducted in adult rodents, and more than thirty of these studies have resulted in mixed outcomes. Effects were or were not detected within a wide range of doses (0.0002 to 960 mg/kg of body weight (bw)) [9,10], exposure periods (6 days–48 weeks) [11,12], administration routes (drinking water, gavage, subcutaneous, intraperitoneal injection) [12,13,14,15], and species of rats (Wistar, Sprague–Dawley, albino) [16,17,18] or mice (Kunming, Pzh:SFIS outbred laboratory, CD-1 mice, Swiss-albino mice, ICR mice, C57BL/6 mice) [12,19,20,21,22,23]. Briefly, in BPA-treated animals, absolute and/or relative reproductive organ weights were decreased [9,10,18,24,25,26,27], or the testes weights were even increased [11]. In some BPA-exposed rodents, a smaller seminiferous tubule diameter [10,26,28], a lower epithelial height of seminiferous tubules [11,16,29], or higher numbers of apoptotic cells [19,21,30,31] were detected. Histopathologically, a decrease in the number of Leydig cells [10,24], the degeneration of Leydig cells and germ cells [27], a reduced number of germ cells [19,21], germ cell exfoliation [12,28], necrosis of germinal epithelium [32], and cytoplasmic vacuolation [29] were reported in the testes of examined animals. Regarding sperm quality and quantity, BPA was reported to decrease epididymal sperm motility, sperm count, sperm production, sperm reserves, and sperm transit time, and to increase sperm fragility and sperm DNA damage [9,14,17,29,33,34]. Furthermore, BPA disturbed the pro-oxidant–antioxidant balance of testicular and epididymal tissue of adult rats [9,17,18,35].

Only two in vivo studies were performed on mammals other than rodents; in one experiment, these animals were common marmosets [36], and in the other, goats [37]. In the latter study, adult male goats were administered BPA (25 mg/kg) as a positive control for the main substance studied—a plant extract, *Ipomoea carnea*. The only lesion noted in BPA-exposed bucks was vacuolar degeneration in the rete testis and the decreased integrity of the plasma membrane of the spermatozoa [37].

Uncertainties remain regarding the toxic potential of BPA on male reproduction in mammals and the relevance of the experimental data to humans. In addition, the experimental data are mainly from specific rodent species and strains, for which varying sensitivity to estrogenic substances has been reported [38]. Furthermore, the most comparable route of exposure for humans would be dietary, since human exposure to BPA is thought to occur mainly via food [39]. This fact is supported by the results of the study by Guignard et al. [40], in which the absolute bioavailability of BPA was about three times higher after dietary administration than after gavage. Therefore, gavage was not considered a better route of exposure than subcutaneous application of BPA [41].

The aim of this multidisciplinary study was to investigate the effects of long-term dietary exposure to a relatively low dose of BPA on the testes, epididymides, and spermatozoa of pubertal rams. During our two-month experimental study, rams in the treated group were fed a diet containing 25 μg BPA/kg bw/day. The dose was chosen on the assumptions of Guignard et al., who suggested that people could ingest several tens of µg BPA per kg per day [40]. Their assumptions of the daily intake were based on pharmacokinetic studies performed on various animals [42] and the commonly described human plasma BPA concentrations in the range of ng/mL [43]. The second aim of this study was to confirm the internal exposure of rams treated with BPA, as internal exposure is rarely reported. Using our analytical method, developed in a previous study on an Istrian Pramenka sheep [44], we aimed to detect free and total BPA in blood and testicular tissue by enzymatic deconjugation (for total BPA), organic solvent extraction, molecularly imprinted polymer solid-phase extraction (MISPE) clean-up, and high-performance liquid chromatography with fluorescence detection (HPLC-FLU). Blood samples were collected multiple times to evaluate the toxicokinetic profile after the first BPA administration.

## 2. Materials and Methods

### 2.1. Chemicals

Bisphenol A ≥ 99% purity (Merck, Sigma-Aldrich, Darmstadt, Germany) was dissolved in absolute ethanol. Solutions were stored at ambient temperature in sealed amber glass bottles for the entire period of use. All materials used for the solution preparation, sample processing, and assays were either glass or BPA-free plastics.

### 2.2. Animals and Their Environment

The study was conducted on adult Istrian Pramenka rams in a sheepfold at the Infrastructure Centre for Sustainable Recultivation Vremščica of the Veterinary Faculty of the University of Ljubljana, Slovenia. The center is located in a rural area in western Slovenia with a temperate continental climate. The rams were born at the center in the wintertime (birth dates are presented in Supplementary Table S1). They were nine-months old at the beginning of the experiment and weighed 34.5–54 kg. The rams were randomly assigned to two groups, a control group (n = 7) and a treated group (n = 7). Random allocation was performed by physical randomization. The rams were marked with conventional sheep ear tags. A caretaker monitored the rams three times per day. Prior to the experiment, a basic veterinary examination (temperature, respiratory rate, pulse rate, rumination frequency) and a basic blood analysis were performed to ensure that rams were clinically healthy (see Supplementary Tables S2–S4).

Rams were kept indoors, under natural light, temperature conditions (6 to 15 °C), and relative air humidity (45–55%) in pens with wooden and metal grids. One collective pen (2.6 × 4 m) contained the bucks of the control group, and the other pen (2.6 × 4 m) contained the bucks of the treated group. No direct contact was allowed between the animals of the different groups, and separate feed and water supplies were provided for each group. Animals were housed individually during administration and sampling.

Water and hay were available ad libitum, and each morning the rams received vegetable pellets (Schafkorn Lac, Unser Lagerhaus Warenhandels Ges.m.b.H). Feeding management was the same for both groups of rams.

We tested for BPA contamination in water, hay, and pellets, and BPA was not detected in any of the samples. The pens and individual stalls, common water supply (50 L enamel pot), and feeding containers (stainless steel bowls) were all made of either wood, enamel, or stainless-steel to minimize background levels of BPA.

Both groups of rams were euthanized at 11 months of age. The experiment lasted 64 days, which is sufficient for the entire duration of spermatogenesis and for the transition of sperm in the *ductus deferens* in rams. Before euthanasia, rams were premedicated with xylazine (2 mL Xylased 5%, Chanelle Pharmaceuticals Ltd., Loughrea, Ireland, i/v) and euthanized after seven to ten minutes with pentobarbital (Exagon, Richter Pharma, Wels, Austria; 2 mL/10 kg bw).

All animal procedures were performed in accordance with ethical standards and approved by the Administration of the Republic of Slovenia for Food Safety, Veterinary Sector and Plant Protection; approval numbers U34401-3/2015/8 and U34401-3/2015/17. All procedures involving animals and the experiment were in accordance with the Slovenian Animal Protection Act [45], Council Directive [46], and ethical principles.

### 2.3. BPA Exposure Protocol

The treated group received a daily dose of 25 µg BPA/kg in the diet. Approximately 1 mL of BPA solution in absolute ethanol was applied to the pellet ration to obtain the single dose of 25 µg/kg body weight, and was administered with the morning feeding of pellets (550 g) that lasted approximately 15 min. A caretaker ensured that rams ate the entire meal each day. The rams were weighed once a week and the administered volume was adjusted to the last recorded body weight. The control group received 1 mL of ethanol without BPA, applied and administered similarly to the BPA-treated group.

Fresh BPA solution was prepared five times during the experiment, and the stability of BPA in ethanol at concentration of 2 mg/mL was proved over 18 days in our previously performed experiment on BPA toxicity in aquaculture.

### 2.4. Sampling Protocols

#### 2.4.1. Blood Plasma

On the first day of the experiment, blood sampling was performed after BPA administration. Blood samples were collected from the rams at time 0 (before the first administration), and at 0.083, 0.16, 0.33, 0.5, 1, 2, 4, 6, and 8 h. The sampling time began when the rams had consumed the entire portion of the pellets.

Blood samples from the jugular vein were collected in heparinized glass vacuum tubes, cooled to 4 °C, and transported to the laboratory where the blood plasma was separated by centrifugation at 2640× *g* for 15 min. Plasma was transferred to polypropylene tubes (PP) and stored. Plasma samples were frozen at −20 °C until analysis. Blank blood plasma samples were collected from rams before the start of the experiment to provide a baseline for analysis. To avoid contamination with BPA during sampling, glass vacuum tubes were used for blood collection.

#### 2.4.2. Gross Morphology and Processing of Organs

After euthanasia, rams were weighed; then, the left and right testes were removed, weighed, and sampled, and the epididymides were removed.

Cross sections were made in both testes to the approximate midpoint for rapid fixation. After fixation in Bouin’s fixative for six hours, a 1 cm-thick block of tissue was excised and fixed for an additional 24 h, rinsed in 70% ethanol, further fixed in neutral phosphate-buffered 4% formaldehyde (10% formalin), further processed, embedded in paraffin wax, sectioned, and stained with a hematoxylin and eosin (H&E) stain and a periodic acid–Schiff (counterstained with hematoxylin) (PAS-H) stain. The complete right epididymis was fixed in 10% buffered formalin, processed, embedded in paraffin, sectioned, and stained with H&E.

The left epididymis was excised, ligated at the *ductus deferens*, individually packed in polyethylene bags, placed on ice, and immediately transported in an ice chest to the laboratory of the Clinic for Reproduction and Large Animals of the Veterinary Faculty of Ljubljana for further processing. After the testes and epididymides were sampled, a complete necropsy was performed on each ram.

### 2.5. Blood Plasma BPA Determination

Samples of blood plasma were analyzed for the presence of free (aglycone) and total (sum of free and conjugated) BPA. Total BPA was determined in free form by enzymatic deconjugation of the glucuronide bond. Blood plasma samples were extracted and prepared for high-performance liquid chromatography (HPLC) analysis, as described by Šturm et al. [44]. The HPLC analysis, quality assurance procedures, validation of BPA analysis, and performance characteristics of BPA analysis are described in the Supplementary Material S3.

### 2.6. Toxicokinetics

Toxicokinetic analyses were performed with EquivTest/PK software (Statistical Solution Ltd., Cork, Ireland). The plasma concentration time course from the first BPA administration was analyzed using a non-compartmental approach to obtain the toxicokinetic parameters. The calculated parameters were C_max_, T_max_, k_el_, t_1/2_, AUC, AUMC, MRT, CL, and Vd. The C_max_ was the maximum observed plasma concentration. The T_max_ was the time of the maximum observed plasma concentration. The k_el_ was the terminal slope of the concentration profile in the semi-log plot calculated by linear regression. The t_1/2_ was the elimination half-life, calculated as the ratio between ln(2) and k_el_. The area under the curve to the last concentration higher than LOQ (AUC_t_) was calculated using the linear trapezoidal method until eight hours, and the area under the curve to the infinity (AUC_i_) was sum of the AUC_t_ and the extrapolated part to infinity by the addition of the term C_last_/ k_el_, where C_last_ is the last quantified concentration (8 h in this study). The area under the moment curve (AUMC) was the area under the curve of the product of concentration and time versus time. The mean residence time (MRT) was calculated as the ratio between the AUMC and AUC. The clearance (CL) was calculated as the ratio between the dose and AUC_i_. Volume of distribution (Vd) was calculated as the ratio between clearance and k_el_.

### 2.7. Histopathology of Testes and Epididymides

A histopathological examination of 4 μm-thick tissue sections of formalin-fixed paraffin-embedded (FFPE) samples of testes and epididymides stained with H&E and PAS-H was performed by light microscopy. The examined slides were blinded with regards to the treatment groups. Testes were evaluated in a “stage-aware” manner [47,48,49]. We based the evaluation of the testes and epididymides on the published recommendations of Lanning et al. [50] and Creasy et al. [51]. The endpoints examined in the testes of rams in our study were perivasculitis, Sertoli-only tubules, segmental hypoplasia, vacuolation of Sertoli cells, multinucleated cells, mononuclear infiltrates, sperm retention, sperm head phagocytosis in the basal Sertoli cell cytoplasm, and sperm granuloma, as well as Leydig cells atrophy, Leydig cells hypertrophy/hyperplasia, and Leydig cells vacuolation. In the rete testis, we examined the following endpoints: mononuclear infiltrates, multinucleated cells, mineralization, and fibrosis. The endpoint examined in the epididymis were mononuclear infiltrates, pyknotic sperm, sloughed epithelial cells and sperm granuloma.

Histopathologic changes were described, wherever possible, according to distribution, severity, and morphological character. Severity scores were assigned on a scale of one to five. Grade 1 (minimal changes) corresponds to a histopathologic change ranging from inconspicuous to barely noticeable, but so minor, small, or infrequent as to warrant no more than the least assignable grade. For multifocal or diffusely distributed lesions, this grade was used for processes where less than approximately 10% of the examined tissue was involved. Grade 2 (slight changes) corresponds to a histopathologic change that is a noticeable but is not a prominent feature of the tissue. For multifocal or diffusely distributed lesions, this grade would be used for processes where between approximately 10 and 25% of the examined tissue would be involved. Grade 3 (moderate changes) corresponds to a histopathologic change that is a prominent but not dominant feature of the tissue. For multifocal or diffusely distributed lesions, this grade would be used for processes where between approximately 25 and 50% of the examined tissue would be involved. Grade 4 (marked changes) corresponds to a histopathologic change that is a dominant but not overwhelming feature of the tissue. For multifocal or diffusely distributed lesions, this grade would be used for processes where between approximately 50 and 95% of the examined tissue would be involved. Grade 5 (severe changes) corresponds to a histopathologic change that is an overwhelming feature of the tissue. For multifocal or diffusely distributed lesions, this grade would be used for processes where more than approximately 95% of the examined tissue would be involved.

A study pathologist examined all slides, which were subsequently peer-reviewed by an internationally accredited toxicological pathology diplomate. Furthermore, a pathology working group (PWG) composed of the reviewing pathologist, a study pathologist, and three additional pathologists reviewed the slides, and the final diagnoses for the reviewed lesions represent a consensus of the PWG.

#### Testis Histomorphometry

Epithelial height, tubular diameter, and tubular area were measured in seminiferous tubules in the left testis of rams. Digital images of H&E-stained paraffin sections were taken with a Nikon Microphot-FXA (Nikon Digital SightDS-2M) microscope using a 40× objective lens in almost all cases, except for three tubules from the control group and seven tubules from the treated group, which were too large to be captured using a 40× objective lens. Therefore, a 20× objective lens was used to capture these latter tubules. Images were stored as uncompressed TIFF files, at 1200 × 900 pixels and 0.4 µm per pixel, as red–green–blue (RGB) images with eight bits per channel. A total of 245 round-shaped, straight-cut, seminiferous tubules at the stages VII and VIII (shortly after spermiation) were evaluated. The number of tubules evaluated from the control and treated groups was 116 and 129, respectively. The mean (range) number of seminiferous tubules measured from the control group was 16.6 (15–23) tubules per ram, and from the treated group, 18.4 (14–24) per ram. Morphometric measurements of the area, diameter, and epithelial height of seminiferous tubules were analyzed by semi-automatic image analysis, as described in Spörndly-Nees et al. [7]. The measurements were performed blinded to the outcome.

### 2.8. Semen Analyses

Semen samples were collected using the flotation method from the right *ductus deferens* and the epididymis, which was separated into three parts: head, body, and tail. Semen samples were analyzed for sperm concentration, morphology, motility, and plasma membrane integrity.

In brief, sperm concentration was assessed with an improved Neubauer hemocytometer. Sperm motility was assessed with a computer-assisted semen analyzer (Hamilton Thorne Biosciences, Version 12.3, Beverly, MA, USA) (CASA). Additionally, plasma membrane integrity was assessed using the hypo-osmotic swelling test (HOST). Seminal smears were prepared for Giemsa staining and were assessed for morphology. All the methods were performed as in Premrov Bajuk et al. [52]. Two repeats were included in the semen analyses, except for the CASA analysis, for which three repeats were included.

The endpoints assessed with the CASA were motility, progressive motility, average path velocity (VAP), straight line velocity (VSL), curvilinear velocity (VCL), amplitude of lateral head (ALH), linearity (LIN), and elongation (ELON). The endpoints assessed in the Giemsa-stained slides for morphological abnormalities were normal spermatozoa, head abnormalities, acrosome abnormalities, neck abnormalities, midpiece abnormalities, proximal droplet, mid droplet, distal droplet, and multiple abnormalities. The endpoints assessed with the HOST test were spermatozoa that showed tail swelling or curling and spermatozoa without tail swelling or bending. For both tests, we examined 200 spermatozoa per slide with immersion oil under 1000× magnification.

### 2.9. Statistical Analyses

The statistical analysis was performed using the statistical software R, version 4.0.5 [53]. Differences in the above parameters between the control group and the treated group were tested separately for each variable using univariate hypothesis tests. For numerical variables (i.e., body weight, testis weight, morphometry, sperm parameters) we first used the Shapiro–Wilk test to test whether the data were normally distributed and, if necessary, applied the F-test to compare variances. If normal distribution and equal variance were not rejected, we compared the groups with the two-sample *t*-test; otherwise, the non-parametric Wilcoxon rank sum test was applied. To compare histopathological endpoints, we used Fisher’s exact test. Due to multiple comparisons, we adjusted the *p* values with a Benjamini–Hochberg correction. A *p* value of less than 0.05 was considered statistically significant.

As two rams (one ram from the control group and one ram from the treated group) were deemed in a peripubertal stage, we excluded them from statistical analyses of testis and epididymis histopathology, morphometry, and sperm analysis, and presented the results for rams that were sexually mature.

## 3. Results

### 3.1. Rams’ Exposure to BPA

**Figure 1:**
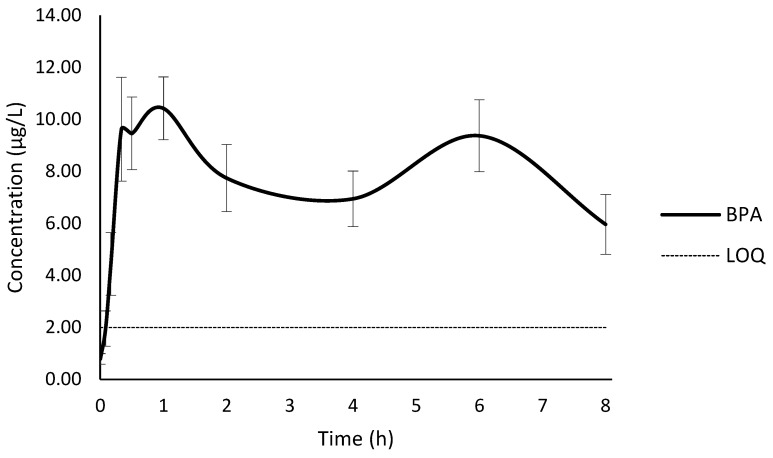
Time course of plasma bisphenol A (BPA) concentration with confidence interval at 95% as error bars and limit quantification (LOQ).

In this study, the C_max_ of BPA was obtained between 20 min and 1 h; in only 1 animal (no. 5), the T_max_ was 6 hours. This confirms the two maxima in the time concentration curve between zero and eight hours (Figure 1). The mean C_max_ was 10.93 µg/L (CV of 17.4%). The k_el_ was calculated from the elimination part of the toxicokinetic curve, but this part started in most of the animals at six hours, after the second maximum; therefore, the accuracy of the extrapolation is low. The mean t_1/2_ was 7.8 h (CV of 27.9%), calculated based on the k_el_.

The AUC from zero to eight hours was 63.4 µg·h/L (CV of 17.2%), and the AUC_i_ based on the k_el_ was 129.4 µg · h/L (CV of 22.3%). The extrapolated part after eight hours was long due to the k_el_ uncertainty. The mean AUMC was 1569.9 µg·h^2^/L (CV of 45.1%) and MRT was 11.8 h (CV of 24.4%), which represents the average time the molecule stays in the body after the first administration of the drug. The mean CL, calculated based on the dose 25 µg/kg bw, was 0.201 L/h/kg bw (CV of 21.1%), and the mean Vd was 2.2 L/kg bw (CV of 25.9%). Calculated toxicokinetic parameters are presented in the Supplementary Table S5.

### 3.2. Mass Measurements

No statistically significant differences were observed in the body weights and testis weights between the control and treated group (Table 1).

### 3.3. Histopathology

#### 3.3.1. Testes

**Figure 2:**
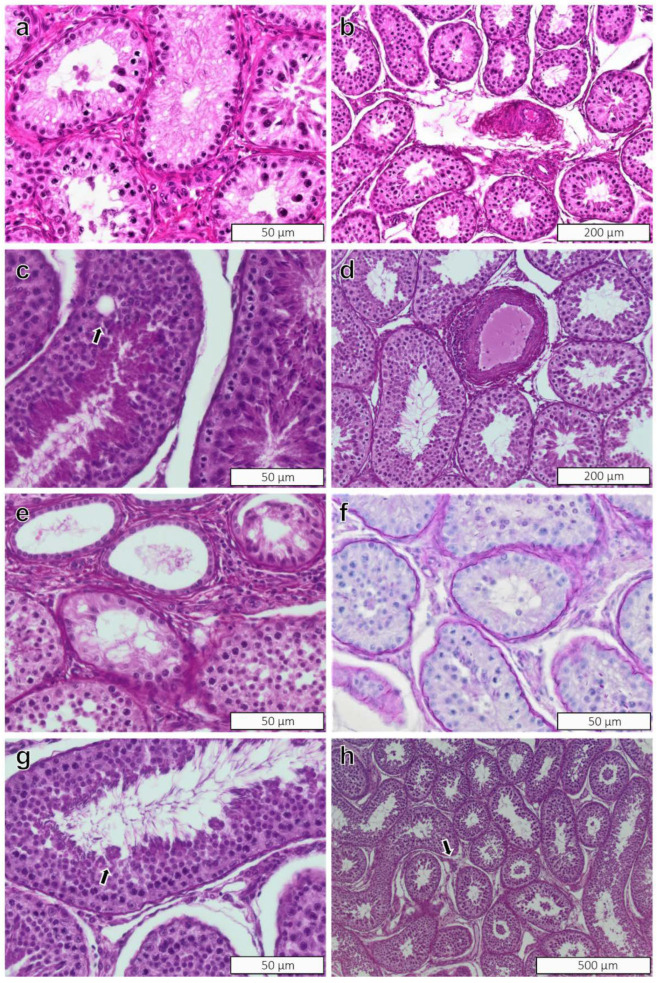
Testes of rams. Spontaneous changes in testis parenchyma and interstitial tissue of rams regardless of treatment group. (**a**) Testis from ram no. 2 with immature testis; (**b**) testis from ram no. 1 with partially mature testis; (**c**) vacuolation of Sertoli-only cell; (**d**) periarteritis; (**e**) hypoplastic tubules near rete testis; (**f**) Sertoli-only tubule; (**g**) multinucleated cells; (**h**) infiltrates of mononuclear cells. H&E stain, with exception of Figure 1f which is stained with PAS-H. Magnification in figures: (**a**,**c**,**e**,**f**,**g**) = ×40; (**b**,**d**) = ×20; (**h**) = ×10.

**Figure 3:**
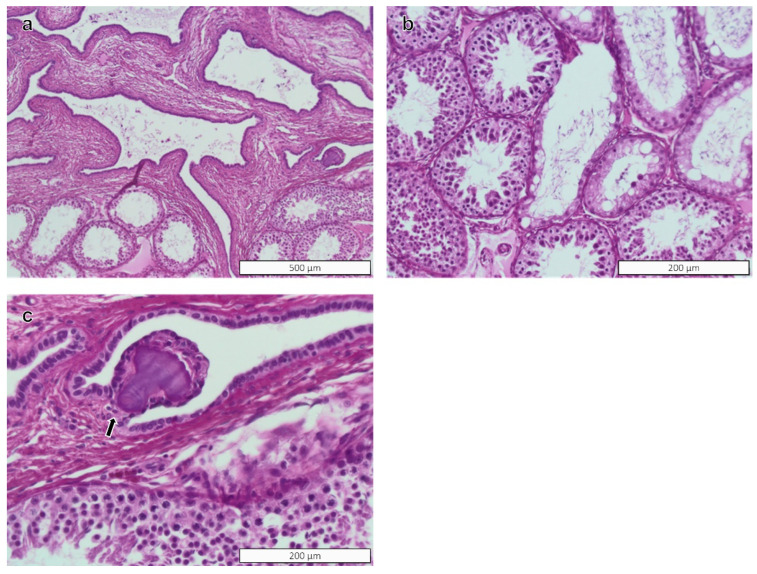
Rete testis of rams. Changes in rete testis of rams. (**a**,**b**) Rete testis and tubules near rete testis (segmental hypoplasia); (**c**) focal mineralization in the mesenchymal tissue of the rete testis. H&E stain. Magnification in figures: (**a**) = ×10; (**b**,**c**) = ×20.

Histopathology findings in the testicular tissue in both groups were mononuclear infiltrates, multinucleated cells, segmental hypoplasia, and vacuolization of Sertoli cells. In addition, focal mineralization was found in the rete testis of a control ram. The severity of these findings was minimal, and none of these findings occurred significantly more frequently in BPA-exposed rams than in rams from the control group (Table 2). All lesions were considered normal alterations that may be encountered in control animals, and were deemed to be within the range of spontaneous alterations. The PWG characterized all findings as non-induced. Images of the most important and/or common changes are presented in Figure 2 for the testicular parenchyma and interstitial tissue, and in Figure 3 for the rete testis.

The status of the animals was considered sexually mature, except for two animals, one control animal (no. 2) and one treated animal (no. 1), which were considered to be in a peripubertal stage.

#### 3.3.2. Epididymides

**Figure 4:**
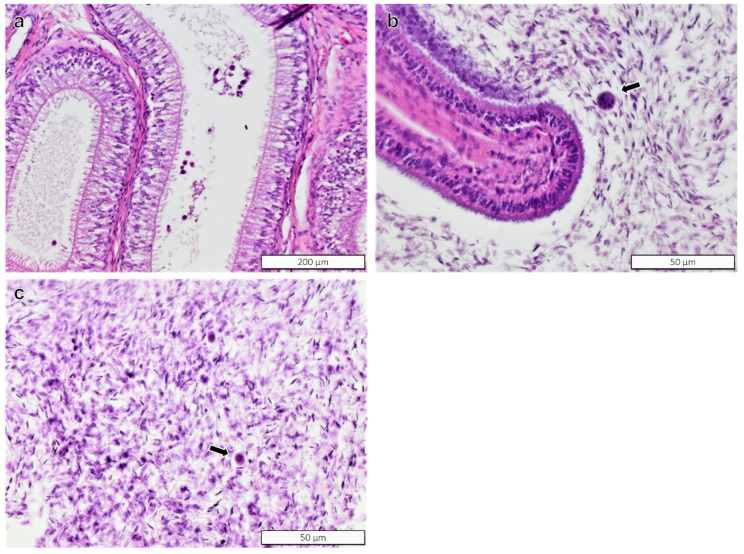
Epididymis of rams. (**a**) Epithelium of the head of the epididymis with cellular detritus; (**b**) pyknotic sperm; (**c**) sloughed cells. H&E stain. Magnification in figures: (**a**) = ×20; (**b**,**c**) = ×40.

Histopathology findings identified in the epididymides were mononuclear infiltrates, pyknotic sperm, intraepithelial fusion cysts, and sloughed epithelial cells. The severity of these findings was minimal, and with exception of pyknotic sperm that were present in BPA-treated rams, no other findings were more prevalent in BPA-exposed rams than in control rams (Table 3). The representative images of the histopathology findings in the epididymides are presented in Figure 4.

### 3.4. Morphometric Measurements

In the exposed group, the seminiferous epithelial height was statistically significantly lower than in the control group (Wilcoxon rank sum exact test, adjusted *p* value = 0.0130). Although the testicular tubule diameter and area were smaller in the treated group compared to control group, there were no statistically significant differences between the two groups of rams (Table 4). Epithelial height, diameter, and area of seminiferous tubules in the testes were measured in stages VII and VIII of spermatogenesis.

### 3.5. Spermatozoa Analysis

There were no statistically significant differences between the control group and the treated group in any of the examined endpoints (Table 5 and Supplementary Material S5).

## 4. Discussion

Our study adds to the discussion of lower-dose toxicity of BPA on male reproduction and challenges the generality of findings in rodent models, using sheep, a small ruminant, as an experimental model. In this study, we did not observe any major signs of altered reproduction in the parameters investigated in rams exposed to 25 μg BPA/kg bw/day via a dietary route, except for the significantly lower epithelial height of the seminiferous epithelium in treated rams.

The dietary route was chosen to mimic realistic exposure to BPA in humans and to minimize stress that would occur with other administration routes. In our study, we chose the dose of 25 μg BPA/kg bw, which is, in many studies, described as a low dose, including those of Consortium Linking Academic and Regulatory Insights on BPA Toxicity (CLARITY-BPA) [54]. However, controversy exists regarding the term ‘’low dose’’, and some researchers stated that this should not be called a low dose as the dose of 25 µg/kg bw corresponds to a daily uptake in humans (adults—60 kg) of 1500 µg of BPA per day [55]. Despite that, in this study, we wanted to consider the most realistic human exposure scenario, with the lowest dose that would still be measurable with our analytical method, to determine the internal exposure of rams with the purpose of detecting possible male reproductive toxicity.

Our results indicate that the dose was not high enough to detect free BPA concentrations; however, we were able to detect total BPA and demonstrate the internal exposure of rams. Interestingly, in our previous study on one Istrian Pramenka sheep [44], the C_max_ for total BPA was 43.46 µg/L, a concentration approximately four times higher than in this study, in which a four-fold-lower dose was administered to the rams, indicating a potential linear toxicokinetic BPA response at concentrations lower than 100 µg/kg. Additionally, the T_max_ for total BPA in our previous study was attained at 0.33 h, that is, in a similar timeframe to this study, where the T_max_ for six rams ranged from 20 min to 1 h.

We observed lower body weights and lower absolute testicular weights in the BPA-treated group, but the differences were not statistically significant. In rodent studies, there is a general divergence in the results of the effect of BPA on body and testicular weight. The lowest and highest doses associated with lower body weight were 0.05 mg/kg bw [26] and 960 mg/kg bw [10], respectively. The lowest and highest doses associated with lower testicular weight were 0.0002 mg/kg bw [25] and 960 mg/kg bw [10], respectively.

The histopathology of testes is acknowledged as the most sensitive endpoint for detecting testicular toxicity in animals [50]. In this study, a histopathological evaluation of testes and epididymides did not reveal any BPA-induced morphological lesions. All lesions were considered normal alterations that may be encountered in control animals, and were deemed to be within the range of spontaneous alterations. Additionally, we did not detect any difference in testes maturation between the groups. The seminiferous tubules were normally developed, with all the germ cells and all the stages of spermatogenesis present. The only exceptions were two rams, one from the control group and the other from the treated group, which were peripubertal. In these two rams, microscopic features, such as hypospermiogenesis, spermatogonial proliferation, and apoptotic and sloughed germ cells were detected in some tubules. The lack of complete spermatogenesis in some tubules may have had an impact on our ability to critically assess the testes as per protocol; thus, these two animals were excluded from further statistical analysis of testes and epididymides histopathological endpoints, and from semen analysis. We found only one study in which comparable animal models, male goats, were exposed to BPA to evaluate male reproductive toxicity. In that study, the bucks were exposed for 120 days to a 1000-fold-higher dose of BPA than in our study, and the only BPA-elicited histopathological lesion was vacuolar degeneration of the rete testis [37]. We analyzed the microphotograph of the lesion in that publication, and our opinion is that this finding is most likely not a vacuolation of the rete testis, but more likely heterotopic fat tissue. In rodent studies, the reported results are divergent; a dose as low as 0.002 mg/BPA/kg/day [31] induced histopathological lesions, whereas in other studies a dose as high as 160 mg/BPA/kg/day did not induce histopathological lesions [10]. In the epididymides, we did not detect any histopathological lesions that were more prevalent in the BPA-exposed group. Only a few investigations on adult experimental animals provided data about lesions in the rodent epididymis, and the main lesion described was a lack of sperm in the epididymal lumina [14,26,43].

In addition to the lack of findings on spermatogenesis and the absence of testicular and epididymal toxicity, sperm morphology, motility, quality, and concentration were not significantly different in BPA-exposed rams compared to the control group in our study. Comparable to the findings of Gotardo et al. [37], in which decreased the plasma membrane integrity of spermatozoa was reported, our study also detected decreased plasma membrane integrity, but it was not statistically significant. Similar to our study, two rodent studies by Qiu et al. [29] and Liu et al. [34] reported the lack of sperm morphology defects due to the BPA treatment, in contrast to one study by Kourouma et al., in which exposure to 2, 10, and 50 mg/kg/bw for 20 days resulted in sperm morphology abnormalities, such as bent tails, coiled tails, detached heads, and double tails [15]. Most rodent studies reported a decline in sperm count [9,13,15,20,27,29,31,32,33,56,57]; nevertheless, in some cases, sperm count was unaffected [23]. Similar results are reported for sperm motility, which was decreased in some cases [9,13,32,57] and unchanged in others [34].

The only observed effect in our study was a statistically significantly lower seminiferous epithelium height in rams exposed to BPA. Our results are in concordance with the lower seminiferous epithelium heights in the studies of Qiu et al. [29] and Ullah et al. [11], but are in contrast with the study of Ogo et al. [16], in which epithelial height of seminiferous tubules of the testes was higher in the BPA-exposed group.

The main limitation of our study is large inter-animal variability in terms of body weights and testis weights, which could mask the subtle BPA effects in histopathology and sperm parameters. Additionally, changes associated with the developing testis in testicular tissues examined histopathologically may have masked effects on the reproductive endpoints, making it difficult to identify endocrine-disrupting effects. The second limitation of the study is the missing data of epididymis weights, as they were not measured due to complicated logistics. The third limitation of the study is that we did not test multiple BPA doses; thus, we cannot address the dose response of BPA in this study. The number of the animals and the duration of our study were limited due to the complexity of the experiment and the use of large food-producing animals. However, in our opinion, there is insufficient evidence in the rodent studies conducted to date that the dose effect is indeed non-monotonic [12,15]. In previously mentioned rodent studies investigating male reproductive toxicity of BPA, the dose response was mostly dose-dependent, and was rarely equal or non-monotonic [12,15,22,32]. Even in the two-generation CLARITY-BPA studies, no non-monotonic dose response was found. In fact, both studies concluded that rat testes and spermatozoa were insensitive to oral BPA exposure over a wide dose range (from 2.5 to 25,000 μg of BPA/kg bw) [58,59].

In conclusion, our results demonstrate that BPA did not arrest the pubertal development of the rams, and did not cause overt toxicity. Additionally, this manuscript presents the partial results of a larger study, in which dietary BPA exposure on femoral morphology, metabolism, mineral content, and biomechanical behavior in young rams was studied [60] and in which, coincidently, no greater effects on bones were detected. Regardless of the absence of effects in this study, further studies with a larger number of animals and, perhaps, a longer duration would be required to confirm our findings.

## Figures and Tables

**Table 1:** Mass measurements of final body weights and testis weights of rams in the control group and the treated group exposed to 25 μg of BPA per kg of body weight per day. The mean values and standard deviations (SD) are reported.

	Control (n = 7) Mean ± SD	Treated (n = 7) Mean ± SD
Body weight (kg)	54.2 ± 7.4	52.6 ± 4.1
Testis, left (g)	150.4 ± 74.1	120.8 ± 27.2
Testis, right (g)	150.8 ± 69.3	118.4 ± 27.2

**Table 2:** Incidence of histopathological findings in right and left testes and rete testis of control and BPA-treated rams. Only mild lesions were detected in every endpoint examined.

	Right Testis		Left Testis		Rete Testis		
Endpoint Examined	Control Group	Treated Group	Control Group	Treated Group	Endpoint Examined	Control Group	Treated Group
Perivasculitis	0/6	0/6	0/6	1/6	Mineralization	1/6	0/6
Sertoli-only tubules	0/6	0/6	0/6	0/6	Multinucleated cells	1/6	0/6
Segmental hypoplasia	1/6	1/6	1/6	0/6	Mononuclear infiltrates	0/6	0/6
Vacuolation of Sertoli cells	0/6	0/6	1/6	0/6	Fibrosis	0/6	0/6
Multinucleated cells	2/6	1/6	4/6	1/6			
Mononuclear infiltrates	5/6	5/6	4/6	6/6			
Sperm retention	0/6	0/6	0/6	0/6			
Sperm head phagocytosis in the basal Sertoli cell cytoplasm	0/6	0/6	0/6	0/6			
Leydig cells atrophy	0/6	0/6	0/6	0/6			
Leydig cells hypertrophy/hyperplasia	0/6	0/6	0/6	0/6			
Leydig cell vacuolation	0/6	0/6	0/6	0/6			

**Table 3:** Incidence of histopathological findings in epididymides of control and BPA-treated rams. Only mild lesions were detected in every endpoint examined.

	Endpoint Examined	Incidence of Findings in Control Rams	Incidence of Findings in Treated Rams
Head of epididymis	Mononuclear infiltrates	3/6	4/6
	Pyknotic sperm	0/6	0/6
	Sloughed epithelial cells	0/6	0/6
	Intraepithelial fusion cysts	0/6	1/6
	Sperm granuloma	0/6	0/6
Body of epididymis	Mononuclear infiltrates	4/6	5/6
	Pyknotic sperm	0/6	0/6
	Sloughed epithelial cells	0/6	0/6
	Intraepithelial fusion cysts	0/6	0/6
	Sperm granuloma	0/6	0/6
Tail of epididymis	Mononuclear infiltrates	2/6	3/6
	Pyknotic sperm	0/6	2/6
	Sloughed epithelial cells	0/6	1/6
	Intraepithelial fusion cysts	0/6	0/6
	Sperm granuloma	0/6	0/6

**Table 4:** Morphometry of young adult rams exposed to 25 μg of BPA/kg bw/day compared to control rams. The mean values and standard deviations (SD) are reported.

	Control (n = 6) Mean ± SD	Treated (n = 6) Mean ± SD
Seminiferous epithelial height (μm)	58.44 ± 6.97	48.71 ± 2.34
Seminiferous tubule diameter (μm)	92.28 ± 9.04	89.48 ± 6.35
Seminiferous tubular area (mm^2^)	26,988 ± 5301	25,255 ± 3453

**Table 5:** Concentration, HOST, progressive motility, and normal morphology of ram spermatozoa after two months of treatment vs. non-treatment. The mean values and standard deviations (SD) are reported.

	Head of Epididymis		Body of Epididymis		Tail of Epididymis		*Ductus deferens*	
Parameter	Control (n = 6)	Treated (n = 6)	Control (n = 6)	Treated (n = 6)	Control (n = 6)	Treated (n = 6)	Control (n = 6)	Treated (n = 6)
Concentration (×10^8^)	1.2 ± 0.9	0.9 ± 0.3	1.4 ± 1.6	1.1 ± 0.7	6.4 ± 1.6	4.4 ± 0.4	0.4 ± 0.4	0.3 ± 0.2
HOST (% of live sperm)	54 ± 6	56 ± 11	64 ± 9	64 ± 11	71 ± 8	67 ± 8	61 ± 7	56 ± 17
Motility (%)	4.6 ± 5.2	1.1 ± 0.6	25.7 ± 15.1	24.1 ± 22.1	96.1 ± 1.1	84.7 ± 16.4	78.7 ± 27.5	55.3 ± 28.6
Progressive motility (%)	0.2 ± 0.4	0 ± 0	2.9 ± 3.5	3.8 ± 3.7	35.1 ± 5.1	30.4 ± 14.8	29.1 ± 11.3	22 ± 16.6

## Data Availability

The data presented in this study are available on request from the corresponding author.

## Supplementary Materials

The following supporting information can be downloaded at: https://www.mdpi.com/article/10.3390/toxics10050224/s1, Supplementary Table S1: Birth dates of rams; Supplementary Tables S2–S4: Parameters of a basic veterinary examination and blood analysis of rams prior to the experiment; Supplementary Material S3: HPLC analysis of blood plasma samples with quality assurance procedures, validation of bisphenol A (BPA) analysis, and performance characteristics of BPA analysis [61,62]; Supplementary Table S5: Toxicokinetic parameters of rams exposed to the first dietary BPA administration; Supplementary Material S5: Results of spermatozoa analysis of rams.
