# Supplementary material for: Basic Exploratory Study of Bisphenol A (BPA) Dietary Administration to Istrian Pramenka Rams and Male Toxicity Investigation
Source: Toxics. 2022 Apr 29;10(5):224. doi: 10.3390/toxics10050224 (PMC9143511; doi:10.3390/toxics10050224)
Supplement: Supplementary file 1 [file toxics-10-00224-s001.zip › toxics-1675497-supplementary.pdf]

toxics

Supplementary Materials: Basic Exploratory Study of Bisphenol A (BPA) Dietary Administration to Istrian Pramenka Rams and Male Toxicity Investigation

Sabina Šturm, Klaus Weber, Primož Klinc, Ellinor Spörndly-Nees, Azadeh Fakhrzadeh, Tanja Knific, Andrej Škibin, Věra Fialová, Yoshimasa Okazaki, Tanja Razinger, Jürgen Laufs, Robert Kreutzer, Milan Pogačnik, Tanja Švara, Vesna Cerkvenik-Flajs

S1. Birth Dates of Rams

Table S1. Birth dates of rams included in the experiment.

	Ram Number	Birth Date
Control group		
	10	February 12.
	9	January 27.
	13	January 19.
	2	January 27.
	3	January 29.
	6	January 26.
	14	January 30.
Treated group		
	4	January 27.
	1	January 27.
	7	January 26.
	12	January 30.
	8	January 29.
	5	January 23.
	11	January 27.

S2. Parameters of a Basic Veterinary Examination and Blood Analysis of Rams Prior to the Experiment

Table S2. Results of clinical examination of the rams prior to the experiment.

	Ram Number	Body Temperature (°C)	Pulse Rate (/min)	Breathing Frequency (/min)	Rumination Frequency (/5 min)
Control group					
	10	40.4	68	40	2
	9	39.4	68	32	2
	13	39.6	68	32	2
	2	39.8	68	24	2
	3	39.9	68	32	2
	6	39.6	68	36	2
	14	40.4	68	36	2
Treated group					

4	39.9	68	39.9	2
1	39.9	68	39.9	2
7	39.7	68	39.7	2
12	39.3	68	39.3	2
8	39.8	68	39.8	2
5	39.9	68	39.9	2
11	39.5	68	39.5	2

toxics MDPI

Table S3. Haematology of the rams prior to the experiment.

Ram number	Erythrocytes (x 10^{12}/L)	Hb (g/dL)	MCV (fL)	Ht (%)	Leukocytes (x 10^{9}/L)	MCH (pg)	MCHC (g/dL)	Platelets (x 10^{9}/L)	Neutrophils (%)	Eosinophils (%)	Basophils (%)	Lymphocytes (%)	Segmented neutrophils (%)	Monocytes (%)
Control group														
10	12.06	12.1	30	35.9	6.6	10.0	33.6	417	40	0	0	59	0	1
9	11.32	11.5	29	33.4	7.0	10.2	34.5	455	32	0	0	68	0	0
13	11.80	12.7	30	34.8	10.0	10.7	36.4	504	23	1	0	75	1	0
2	13.20	13.2	30	40.2	4.7	10.0	32.9	698	48	1	0	49	0	2
3	11.86	12.0	30	36.1	9.3	10.1	33.3	538	29	3	0	67	0	1
6	13.40	13.1	32	42.2	7.7	9.8	31.1	563	40	1	0	59	0	0
14	13.73	13.1	31	42.2	8.9	9.5	31.0	301	33	1	0	64	0	2
Treated group														
4	12.62	12.7	30	37.7	7.8	10.1	33.7	489	40	1	0	58	0	1
1	12.23	12.3	31	37.5	9.0	10.1	32.9	550	41	2	0	57	0	0
7	12.47	12.3	31	38.3	6.2	9.9	32.1	707	61	1	0	37	0	1
12	10.98	10.8	31	34.4	10.1	9.8	31.4	558	38	4	0	58	0	0
8	12.45	12.5	31	38.5	9.8	10.0	32.4	339	29	1	0	67	1	2
5	13.54	13.1	32	43.8	8.1	9.7	29.8	345	49	1	0	50	0	0
11	13.54	12.4	32	43.2	7.8	9.2	28.8	531	36	0	0	62	0	2

Legend: Hb – hemoglobin, MCV – mean corpscular volume, Ht – hematocrit, MCH – mean corposcular hemoglobin, MCHC – mean corposcular hemoglobin concentration. Haematological analyses were performed utilising the Scil Vet abc Plus (Horiba, Japan) automated haematological analyser. Differential white blood cell count was determined according to the standard procedure; smears were stained with Hemacolor (Merck, Darmstadt, Germany) and manually counted via microscopic examination. Blood samples for the haematological analyses were taken on 13. 10. 2017, haematological analyses were performed on 13. 10. 2017 and 18. 10. 2017.

Table S4. Biochemistry results of the rams prior to the experiment.

	Ram Number	Urea (mmol/L)	Ca (mmol/L)	PO_4 (mmol/L)	Creatinine (μmol/L)	Cholesterol (mmol/L)	Triglycerides (μmol/L)	AST (μkat/L)	GGT (μkat/L)
Control group									
	10	4.5	2.6	2.69	64	1.8	0.4	1.83	0.88
	9	4.6	2.58	2.69	49	2.1	0.4	2.29	0.72
	13	5.2	2.79	2.48	80	2.1	0.3	2.41	0.93
	2	5.9	2.35	2.72	52	1.7	0.2	1.66	1.09
	3	5.1	2.58	3.05	54	1.5	0.5	2.28	0.98
	6	5.7	2.58	3.06	63	1.8	0.3	2.08	0.69
	14	6.2	2.62	2.83	56	1.7	0.2	2.95	1
Treated group									
	4	6	2.53	3.23	55	2	0.4	2.85	1.19
	1	4.8	2.48	3.93	62	2.1	0.3	2.6	0.92
	7	4.5	2.48	2.83	53	1.7	0.4	2.62	1.07
	12	4.2	2.4	3.25	53	1.8	0.5	2.08	1.14
	8	5.4	2.48	2.8	51	1.7	0.3	3.05	1
	5	5.9	2.56	2.53	69	2.7	0.3	2.54	0.97
	11	6.7	2.53	3.21	75	2.1	0.5	2.56	0.89

Legend: Ca – calcium, PO_4 – inorganic phosphate, AST – aspartate aminotransferase, GGT – gamma glutamyl transferase. For biochemistry analysis, automatic chemistry analyzer (Olympus Corp., Hamburg, Germany) was utilized.

S3. HPLC Analysis of Bisphenol A (BPA) in Blood Plasma Samples with Quality Assurance Procedures, Validation and Performance Characteristics

HPLC analysis

A 50 μL aliquot of the blood plasma samples was taken for the high-performance liquid chromatography (HPLC) analysis. A Hypersil GOLD C18 (150 × 4.6 mm, 3 μm particle size) analytical column was used which was protected by Hypersil GOLD 3μ drop in guard cartridges (Thermo Scientific, Waltham, MA, USA). The chromatographic process was performed at room temperature using a gradient HPLC method. The mobile phase was pumped at a flow rate of 1.0 mL/min and used the two constituents of a mobile phase, i.e., H_2O (constituent A) and MeCN (constituent B) in the following volume ratios: time 0–2 min (35% B), time 2–12 min (gradient 35–50% B), time 12–20 min (50% B), time 20–20.5 min (gradient 50–35% B) and time 20.5–21 min (35% B). The excitation and emission wavelengths of the fluorescence spectrophotometry analysis were set at 230 and 315 nm, respectively [61]. The results were evaluated according to an external standard method using a solvent standard calibration curve, which was constructed by plotting the peak area as a function of the analyte concentration.

Quality assurance procedures

Each sample series consisted of a baseline reagent sample, a baseline matrix sample, the study samples (in duplicate) and the two recovery samples. These were obtained by fortification of the baseline matrix sample with BPA on a reasonable level. Solvent standard calibration curves were constructed from 6 calibration points. The measured replicate study sample concentrations were corrected for the mean recovery rate of the respective

series, for an overall value of a baseline reagent sample, and the mean found concentration was used as a result.

Validation of BPA analysis

Validation was done separately for free and total BPA. Linearity was determined on a standard level by the least squares method, giving the regression and correlation parameters of the calibration lines. Solvent standard concentrations with 6 concentration points per calibration line ranged from 0.5–25 ng/mL in blood plasma. Recovery and intra-laboratory reproducibility of the method were tested by the response of the fortified matrix samples on separate time occasions. For free BPA determination in blood plasma these were fortified at 2 μg/L (n = 8) and for total BPA determination they were fortified at 10 and 20 μg/L (n = 8).

The precision of the methods was evaluated as the standard deviation and the coefficient of variation (CV) of the determined values and was assessed in accordance with the Horwitz coefficients (CV_H) according to Commission Decision 2002/657/EC [62]. The limit of quantification (LOQ) value was determined as the lowest analyte content for which the method proved acceptable in terms of recovery and repeatability.

Performance characteristics of BPA analysis

The analytical HPLC methodology used demonstrated good linearity, by the correlation coefficients' "R-squared" values of the solvent standard calibration lines of >0.998. The mean recovery values for determination of free BPA (fortification level of 2 μg/L) and total BPA (fortification levels of 10 and 20 μg/L) were 82.9% and 50.1%, respectively. The within-laboratory reproducibility of the measurements of the free and total BPA, represented by the CV values, were 10.5%, and 19.3%. The estimated LOQ values for determination of free and total BPA were 0.5 and 2 μg/L, respectively.

S4. Toxicokinetic Parameters of Rams Exposed to the First Dietary BPA Administration

Table S5. Toxicokinetic parameters of rams exposed to the first dietary BPA administration.

Ram No.	BW (kg)	C_{max} (μg/L)	T_{max} (h)	k_{el} (h-1)	$t_{1/2}$ (h)	AUC_t (μg · h/L)	AUC_i (μg · h/L)	AUMC (μg · h2/L)	MRT (h)	Cl (L/h/kg)	Vd (L/kg)
**4**	38.0	9.52	1.00	0.104	6.7	54.4	98.9	993.2	10.0	0.253	2.43
**1**	43.5	8.89	0.50	0.067	10.4	55.8	167.6	2802.6	16.7	0.149	2.24
**7**	44.5	10.69	1.00	0.078	8.9	58.5	119.3	1493.4	12.5	0.209	2.68
**12**	43.0	13.86	0.33	0.104	6.7	72.8	129.2	1279.0	9.9	0.194	1.86
**8**	47.0	9.15	1.00	0.086	8.1	51.1	99.9	1152.1	11.5	0.250	2.91
**5**	44.5	11.70	6.00	0.171	4.1	73.5	122.1	972.8	8.0	0.205	1.20
**11**	42.0	12.72	0.50	0.072	9.7	77.9	169.1	2296.3	13.6	0.148	2.06
**n**	7	7	7	7	7	7	7	7	7	7	7
**Mean**	43.2	10.93	1.48	0.097	7.8	63.4	129.4	1569.9	11.8	0.201	2.20
**SD**	2.8	1.90	2.01	0.036	2.2	10.9	28.8	707.3	2.9	0.042	0.57
**CV (%)**	6.4	17.4	136.5	36.6	27.9	17.2	22.3	45.1	24.4	21.1	25.9

n – number of rams, SD – standard deviation, CV – coefficient of variation, C_{max} – maximum plasma concentration, T_{max} – time of maximum plasma concentration, k_{el} – elimination rate constant, $t_{1/2}$ – elimination half-life, AUC – area under the curve, AUMC – area under the moment curve, MRT – mean residence time, Cl – clearance, Vd – volume of distribution.

S5. Results of Spermatozoa Analysis of Control and Treated Rams

Table S6. Results of spermatozoa analysis of the head of epididymis (mean ± standard deviation reported).

Examined Parameter	Control Group (n = 6)	Treated Group (n = 6)

CASA fresh		
ALH (μm)	2.108 ± 3.464	1.733 ± 3.668
ELO (%)	42.917 ± 13.922	51.583 ± 8.879
LIN (%)	43.167 ± 18.549	44.833 ± 13.615
Motility (%)	4.583 ± 5.229	1.083 ± 0.585
Progressive motility (%)	0.167 ± 0.408	0.000 ± 0.000
VAP (μm/sec)	38.292 ± 16.102	43.900 ± 9.672
VCL (μm/sec)	55.383 ± 27.356	62.208 ± 10.349
VSL (μm/sec)	29.025 ± 15.390	27.067 ± 10.947
HOST test (%)		
Dead	45.917 ± 6.320	44.000 ± 10.863
Live	54.083 ± 6.320	56.000 ± 10.863
Morphology of spermatozoa (%)		
Deformed acrosome	0.167 ± 0.408	0.000 ± 0.000
Detached acrosome	0 ± 0	0 ± 0
Acrosome in detachment	0 ± 0	0 ± 0
Distal droplet	11.000 ± 3.847	17.167 ± 7.885
Head abnormalities	0.5 ± 0.837	0.0 ± 0.000
Medial droplet	4.500 ± 3.564	5.333 ± 2.338
Mid-piece abnormalities	0 ± 0	0 ± 0
Multiple abnormalities	3.667 ± 3.938	3.500 ± 2.429
Neck abnormalities	0 ± 0	0 ± 0
Normal	13.000 ± 2.759	14.667 ± 7.633
Proximal droplet	66.333 ± 7.992	57.167 ± 14.959
Tail abnormalities	0.833 ± 0.408	2.167 ± 2.994
Spermatozoa concentration	122645833 ± 88744733	88541667 ± 26597159

Table S7. Results of spermatozoa analysis of the body of epididymis (mean ± standard deviation reported).

Examined Parameter	Control Group (n = 6)	Treated Group (n = 6)
CASA fresh		
ALH (μm)	7.050 ± 2.230	6.683 ± 1.481
ELO (%)	52.25 ± 3.205	48.25 ± 2.603
LIN (%)	39.250 ± 4.967	39.667 ± 4.143
Motility (%)	25.667 ± 15.075	24.083 ± 22.101
Progressive motility (%)	2.917 ± 3.513	3.750 ± 3.725
VAP (μm/sec)	61.183 ± 9.764	73.4167 ± 12.663
VCL (μm/sec)	102.383 ± 20.275	125.550 ± 30.341
VSL (μm/sec)	40.692 ± 8.789	51.717 ± 14.516
HOST test (%)		
Dead	36.167 ± 9.004	35.917 ± 10.938
Live	63.833 ± 9.004	64.083 ± 10.938
Morphology of spermatozoa (%)		
Deformed acrosome	0.167 ± 0.408	0.000 ± 0.000

Detached acrosome	0.333 ± 0.516	0.333 ± 0.516
Acrosome in detachment	0.500 ± 0.837	0.167 ± 0.408
Distal droplet	28.833 ± 17.314	36.000 ± 16.601
Head abnormalities	0.167 ± 0.408	0.333 ± 0.516
Medial droplet	1.833 ± 1.472	3.333 ± 2.066
Mid-piece abnormalities	0 ± 0	0 ± 0
Multiple abnormalities	27 ± 12.361	16 ± 4.195
Neck abnormalities	0.333 ± 0.816	0.000 ± 0.000
Normal	35.833 ± 11.392	34.167 ± 10.245
Proximal droplet	2.167 ± 1.329	1.667 ± 0.816
Tail abnormalities	2.833 ± 2.137	8.000 ± 8.414
Spermatozoa concentration	144843750 ± 156341692	112593750 ± 71684680

Table S8. Results of spermatozoa analysis of the tail of epididymis (mean ± standard deviation reported).

Examined Parameter	Control Group (n = 6)	Treated Group (n = 6)
CASA fresh		
ALH (µm)	7.633 ± 0.382	8.142 ± 0.816
ELO (%)	50.917 ± 2.223	49.833 ± 2.787
LIN (%)	41.417 ± 0.917	40.917 ± 5.643
Motility (%)	96.083 ± 1.068	84.667 ± 16.440
Progressive motility (%)	35.167 ± 5.125	30.417 ± 14.847
VAP (µm/sec)	97.383 ± 11.800	102.525 ± 11.580
VCL (µm/sec)	171.900 ± 21.052	180.225 ± 20.352
VSL (µm/sec)	72.342 ± 9.281	76.408 ± 11.812
HOST test (%)		
Dead	29.417 ± 7.664	33.000 ± 8.198
Live	70.583 ± 7.664	67.000 ± 8.198
Morphology of spermatozoa (%)		
Deformed acrosome	0 ± 0	0 ± 0
Detached acrosome	0.167 ± 0.408	0.167 ± 0.408
Acrosome in detachment	0.167 ± 0.408	0.333 ± 0.516
Distal droplet	34.5 ± 17.363	25.0 ± 11.628
Head abnormalities	0.333 ± 0.516	0.000 ± 0.000
Medial droplet	0.667 ± 0.816	0.500 ± 0.548
Mid-piece abnormalities	0 ± 0	0 ± 0
Multiple abnormalities	1.167 ± 1.941	1.500 ± 2.074
Neck abnormalities	0 ± 0	0 ± 0
Normal	59.5 ± 17.283	69.0 ± 14.339
Proximal droplet	2.167 ± 2.137	1.333 ± 1.751
Tail abnormalities	1.333 ± 1.506	1.667 ± 1.038
Spermatozoa concentration	644583333 ± 163473367	441458333 ± 37101353

Table S9. Results of spermatozoa analysis of the *ductus deferens* (mean ± standard deviation reported).

Examined Parameter	Control Group (n = 6)	Treated Group (n = 6)
CASA fresh		
ALH (μm)	7.65 ± 0.920	7.05 ± 0.694
ELO (%)	53.000 ± 5.030	53.333 ± 4.389
LIN (%)	41.250 ± 3.921	42.167 ± 4.844
Motility (%)	78.667 ± 27.531	55.333 ± 28.639
Progressive motility (%)	29.083 ± 11.320	22.000 ± 16.601
VAP (μm/sec)	95.15 ± 13.070	85.40 ± 16.087
VCL (μm/sec)	172.275 ± 26.578	151.991 ± 24.841
VSL (μm/sec)	73.233 ± 10.506	68.242 ± 15.490
HOST test (%)		
Dead	38.833 ± 7.488	44.500 ± 16.703
Live	61.167 ± 7.488	55.500 ± 16.703
Morphology of spermatozoa (%)		
Deformed acrosome	0.167 ± 0.408	0.167 ± 0.408
Detached acrosome	0.833 ± 0.408	0.0833 ± 0.204
Acrosome in detachment	0.233 ± 0.572	0.000 ± 0.000
Distal droplet	46.167 ± 6.646	27.583 ± 17.351
Head abnormalities	0.750 ± 0.987	0.167 ± 0.408
Medial droplet	1.000 ± 0.632	0.917 ± 1.114
Mid-piece abnormalities	0.083 ± 0.204	0.083 ± 0.204
Multiple abnormalities	2.667 ± 1.033	1.250 ± 1.172
Neck abnormalities	0.833 ± 1.602	0.667 ± 1.033
Normal	43.750 ± 7.069	63.833 ± 14.386
Proximal droplet	1.083 ± 1.497	0.583 ± 0.801
Tail abnormalities	2.667 ± 2.338	4.667 ± 3.266
Spermatozoa concentration	39694444 ± 38793064	27895833 ± 22256799

CASA: computer-assisted sperm analysis. ALH: amplitude of lateral displacement of the sperm head. ELO: elongation. LIN: linearity. VAP: average path velocity. VCL: curvilinear velocity. VSL: straight velocity. HOST: hypo-osmotic swelling test.
